# Two-Scale Simulation of Drop-Induced Failure of Polysilicon MEMS Sensors

**DOI:** 10.3390/s110504972

**Published:** 2011-05-04

**Authors:** Stefano Mariani, Aldo Ghisi, Alberto Corigliano, Roberto Martini, Barbara Simoni

**Affiliations:** 1 Dipartimento di Ingegneria Strutturale, Politecnico di Milano, Piazza L. da Vinci 32, 20133 Milano, Italy; E-Mails: aldo.ghisi@polimi.it (A.G.); alberto.corigliano@polimi.it (A.C.); martini@imec.be (R.M.); 2 MH Division, STMicroelectronics, Via Tolomeo 1, 20010 Cornaredo, Italy; E-Mail: barbara.simoni@st.com

**Keywords:** polysilicon MEMS, drops and shocks, brittle cracking, multi-scale simulations, finite element analysis

## Abstract

In this paper, an industrially-oriented two-scale approach is provided to model the drop-induced brittle failure of polysilicon MEMS sensors. The two length-scales here investigated are the package (macroscopic) and the sensor (mesoscopic) ones. Issues related to the polysilicon morphology at the micro-scale are disregarded; an upscaled homogenized constitutive law, able to describe the brittle cracking of silicon, is instead adopted at the meso-scale. The two-scale approach is validated against full three-scale Monte-Carlo simulations, which allow for stochastic effects linked to the microstructural properties of polysilicon. Focusing on inertial MEMS sensors exposed to drops, it is shown that the offered approach matches well the experimentally observed failure mechanisms.

## 1. Introduction

Because of their micrometric size and their coupled electro-mechanical operating principles, micro electro-mechanical systems (MEMS) can be exposed to several failure mechanisms. The most relevant and diffused failures, which involve the physical properties of the polysilicon constituting the movable parts of the MEMS, are: stiction [1] and other surface interaction phenomena [2,3]; static and dynamic pull-in [4–6] and cracking [7,8]. All these phenomena lead to, at least a temporary malfunctioning of the sensors, and therefore need to be appropriately accounted for in any reliability analysis of these devices. The interactions of the MEMS with the outer environment in terms of temperature, pressure and moisture content can also represent serious issues [9,10].

In this paper we focus on mechanical failures induced by shocks, and we investigate the effects of drops caused, e.g., by mishandling of portable devices. We assume throughout that mechanical loadings have a strong impact on failure, whereas the electro-mechanical coupling affects it negligibly; hence, outcomes turn out to be predictive in the case of high-*g* loadings, as shown in [11].

Different approaches have been recently proposed to describe the post-impact response of polysilicon MEMS inertial sensors, see e.g., [12–16]. Most of them are built upon beam- and plate-like reduced models of the movable parts of the MEMS (usually, the suspension springs and the seismic plate). As far as loading is concerned, a crude description of its time evolution in terms of sine waves is usually adopted (see [17]); this may possibly lead to over-estimations of the drop heights causing failure.

In [18] we started a numerical investigation of the drop-induced failure phenomenon, highlighting and thereby fully exploiting its multi-scale physics. According to the typical geometry of packaged MEMS, we clearly identified three length-scales: a macroscopic one, comparable to the size of the whole package; a mesoscopic one, at the MEMS level; and a microscopic one, which intrinsically owns a characteristic length on the order of the polysilicon grain size. We then attacked the problem of reliably modelling the failure mechanism, following two slightly different paths, either allowing for microstructural features of the polysilicon morphology [19–21] or not [11,22]. In the former approach (termed three-scale approach), to get insights into the link between polysilicon features and failure mechanism we adopted Monte-Carlo simulations, accounting for stochastic effects (due to e.g., polycrystal morphology and grain boundary, GB properties) at the nm length-scale. In the latter approach (termed two-scale approach) we instead avoided predicting the failure evolution, and just provided tools to localize where cracking might take place. The second approach is more industrially-oriented, since the time-consuming micro-scale Monte-Carlo simulations, aimed at modelling the nucleation and subsequent propagation of cracking, have been dropped.

A cross-validation of the two aforementioned approaches is still missing; in this work we try to provide details pertinent to this partially missing link. As compared to previous analyses, we also adopt at the sensor level an enhanced constitutive model for brittle materials; this model is capable of furnishing objective (*i.e.*, space discretization independent) results in terms of failure mechanism, provided that finite element grids in the failing regions are refined enough to accurately resolve the drop-induced stress field.

The capability of the offered two-scale procedure is assessed by tracking the failure mechanisms experimentally observed in uni-axial commercial off-the-shelf accelerometers like those already investigated in [20,21]. It is here shown that the two-scale approach provides an effective description of the drop-induced crack nucleation and propagation up to percolation, able to highlight the effects of polysilicon on the failure itself. It also furnishes results in agreement with those obtained with the more accurate (but far more expensive in terms of computational costs) three-scale approach. From an industrial perspective, the present approach thus looks promising for routinely modelling in a simple frame the effects of shocks and drops on inertial MEMS.

The remainder of the paper is organized as follows. In Section 2 we present a brief survey of the typical failure mechanisms induced by shock loadings on microsystems. In Section 3 we describe the fundamentals of the multi-scale method developed in our previous works and adopted here, whereas in Section 4 we focus on the constitutive model adopted to describe crack nucleation and propagation in polysilicon. In Section 5 we show results concerning the tracking of the failure mechanism in a uni-axial MEMS accelerometer, subjected to drops featuring different falling orientations. Section 6 presents our concluding remarks on the proposed two-scale simulations.

## 2. A Brief Survey of Shock-Induced Mechanical Failures of MEMS Sensors

Inertial sensors are characterized by massive parts, connected to the die through suspension springs. Under shock loadings, the aforementioned massive components may undergo wild oscillations relative to the die; while in the small displacement (and therefore deformation) regime it is expected that sensor output is proportional to the forcing acceleration, shocks can cause the sensor to exit the linear regime. In order to achieve high sensitivity to the external actions, the suspension springs are typically slender and flexible; the MEMS layout therefore features massive parts (seismic mass) with high stress-carrying capacity, directly linked to slender beams (suspension springs) prone to failure if exposed to shocks.

**Figure 1.**
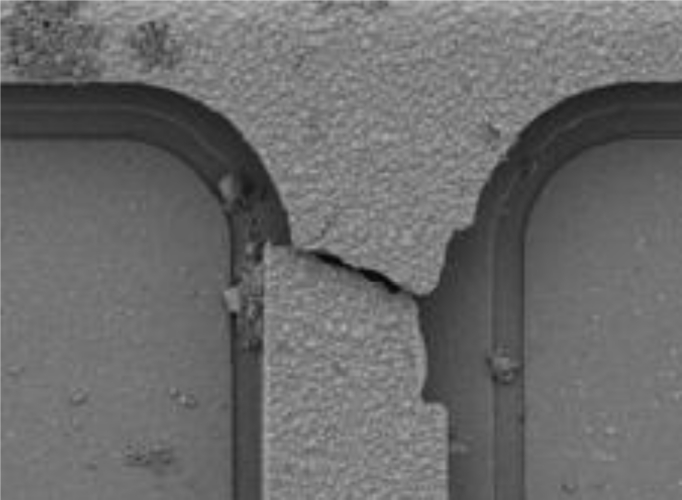
Failure of a suspension spring of a MEMS accelerometer, after [14] (© [2006] IEEE).

**Figure 2.**
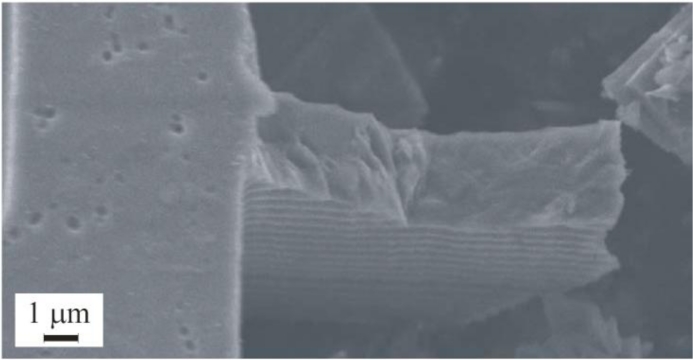
Failure at the connection between anchor and suspension spring of a uni-axial MEMS accelerometer, after [20].

Relevant shock-induced mechanical failures were investigated in [14], where fracture of the suspension springs of a MEMS accelerometer was studied (see Figure 1). In this case failure occurred close to the anchor; such kinds of failure are directly related to the sensor layout, which leads to stress intensification in that region because of the re-entrant corners at the spring-anchor joint. Very similar failure mechanisms were observed in [18,20]; in Figure 2 the typical failure mechanism experienced by a MEMS subjected to laboratory drop tests is depicted, once again showing crack patterns close to the spring-anchor (or spring-plate) joint.

**Figure 3.**
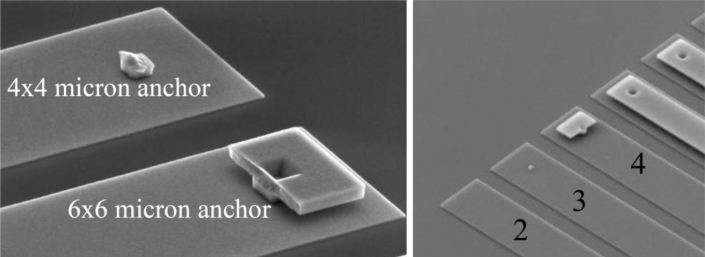
Effect of the anchor size on failure in micro-beams exposed to high-*g* shocks, after [23].

In [23] (see Figure 3), a study on micro-beams exposed to high-*g* shocks, allowed to assess the effect of the anchor size on failure occurrence. Independently of the failure mechanism, *i.e.*, independently of the fact that failure occurs close to or within the anchor, it was observed that polysilicon always fails in region where re-entrant corners amplify the shock-induced stress field.

**Figure 4.**
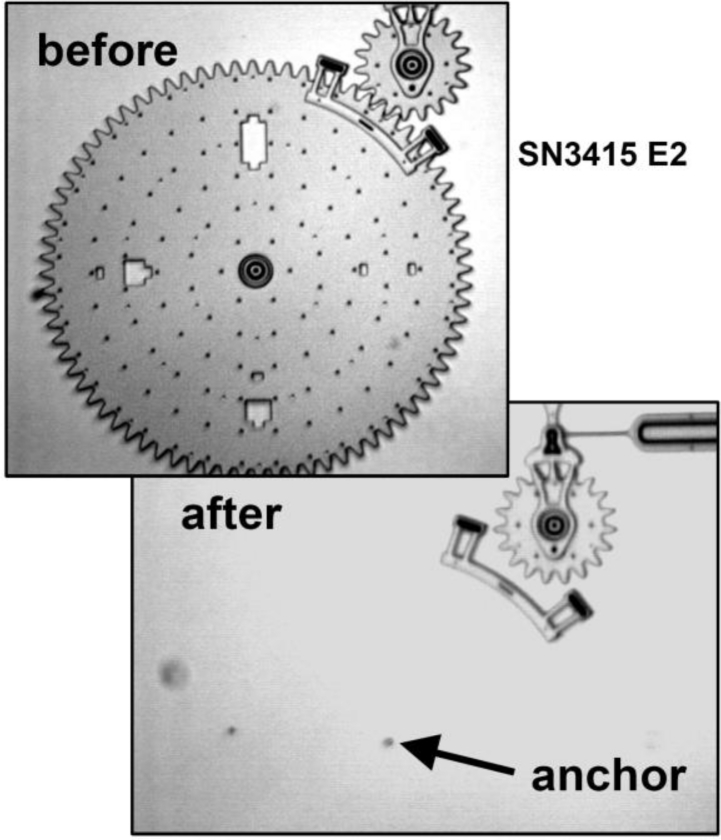
20,000*g* shock-induced failure of the anchor of a micro-gear, after [24] (© [2000] IEEE).

Other microsystems are exposed to shock-induced failures located at or close to the anchor. Tanner *et al.* in [24] showed that micro-gears subjected to a high-*g* loadings (on the order of 10^4^–10^5^ *g*, like those induced by drops, see [18]) may break away from the substrate, as shown in Figure 4.

**Figure 5.**
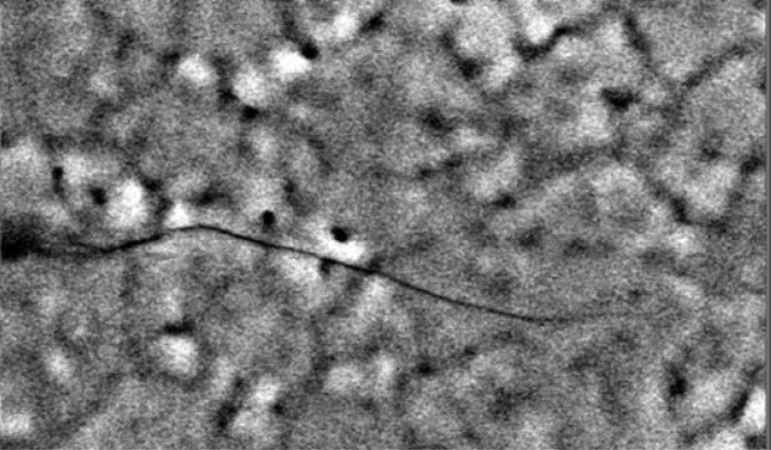
Fatigue-induced crack propagation in a polysilicon film, after [25] (© [2003] IEEE).

**Figure 6.**
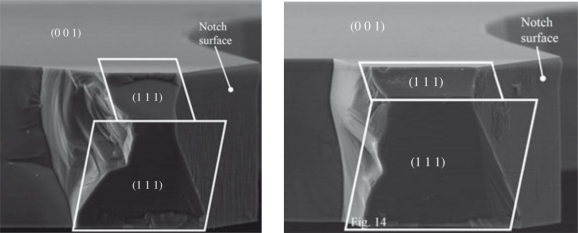
Post-mortem SEM images of the shock-induced failure mechanism in micro-beams, showing crack surfaces aligned with {111} crystal planes, after [26] (© [2008] IEEE).

Even though failures happen under shock loadings, typically exceeding 10^4^ *g*, stochastic effects at the micro-scale may affect the outcomes. This is primarily due to the microstructure of polysilicon films: each microsystem features its own polycrystal morphology in the failing regions, and the overall stress- or shock-carrying capacity turns out to be therefore affected. For instance, Figure 5 shows the path followed by a crack in a microsystem used to assess the fatigue properties of polysilicon [25]; it can be clearly seen that crack kinks almost every time its tip crosses a grain boundary (represented in the figure by dark lines), because of the different orientation of the crystal lattice inside each grain. As shown experimentally in [26], the different atom packing along different crystal orientations causes the crack resistance to be maximum along the {100} crystallographic directions, and minimum along the {111} directions; inside each grain, cracks therefore tend to follow a path dependent on the crystal lattice orientation (Figure 6).

Taking all the aforementioned features into account in numerical simulations requires one to allow for parameters which are not deterministically known, like e.g., crystal orientations and polycrystal morphology; time-consuming statistical investigations thus appear compulsory. Here we aim to propose a computational approach to the study of failure mechanisms, which neglects micromechanical, *i.e.*, polycrystalline details. This method is then validated against stochastic multi-scale (three-scale) simulations.

## 3. Multi-Scale Simulations

Standard finite element simulations of shock-induced crack propagation in packaged MEMS, based on homogeneously refined space discretizations of the whole device, would be too expensive. This is caused by the difference between the size of the whole package (typically a few millimeters) and the length of the zone where dissipative micro-cracking phenomena, which precede the formation of a dominant crack and sensor failure, take place (tens of nm in the case of silicon). Within this latter zone (commonly termed process zone, PZ), the stress and strain fields have to be accurately resolved to get objective, mesh-independent results.

**Figure 7.**
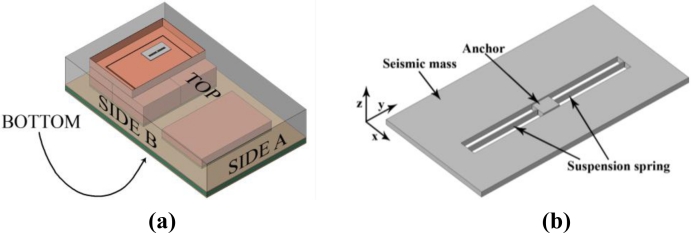
Sketch of the studied device: **(a)** macro-scale model of the whole package; **(b)** meso-scale model of the uniaxial MEMS accelerometer.

In order to reduce the computational burden, in [18–22] a multi-scale framework was suggested and adopted, where the dynamics of the whole MEMS was tracked to eventually foresee the propagation of cracks in the failing region(s). Here, by exploiting the sensor geometry sketched in Figure 7, we decompose the problem into a microscopic and a mesoscopic ones. In macro-scale analyses the whole device [Figure 7(a)], subjected to shock loads, is modeled. At this length-scale the dynamics of the MEMS is disregarded, since its mass is so small (as compared to the mass of the whole device) that inertial forces associated to its motion can not affect the package dynamics. In meso-scale analyses we instead model the dynamics of the MEMS [Figure 7(b)], as induced by the stress waves propagating inside the package and impinging upon the anchor point. Micro-scale analyses, termed this way since they focus on the effects of the polysilicon microstructure on failure, are not considered in the two-scale approach here investigated.

In former investigations, see [18,22], we showed that meso-scale analyses allow to recognize the regions of the MEMS sensor where the stress field attains a critical threshold in terms of maximum principal stress: according to a Rankine strength criterion for brittle materials, these regions are expected to crack. Formerly, we did not try to also track the resulting failure mechanisms.

In [19–21] we also handled micro-scale analyses to provide insights into the failure mechanism; a Monte-Carlo methodology was adopted to properly account for stochastic features linked to the granular nature of polysilicon, and to eventually pin down the statistics of failure in terms of crack pattern and crack velocity. To attain convergence of the aforementioned statistics, at least 100 realizations of the polysilicon morphology in the failing region were considered, each one featuring its own GB geometry and crystal lattice orientations. For this reason, micro-scale analyses turn out to be the most time-consuming step of the multi-scale analysis.

In this work we merge the features of the aforementioned approaches, and simulate the whole failure mechanism without accounting for the actual polysilicon morphology in the failing region. In fact, in [21] we showed that, once details of the polycrystalline morphology are appropriately treated as random variables at the micro-scale, the overall behavior of the failing film does not differ much from that of a homogeneous, quasi-isotropic material; hence, micromechanical features result to be irrelevant at the meso-scale.

Crack propagation in the failing regions is here simulated via a smeared crack model, see [27]. From the physical point of view, it is assumed that cracking is locally incepted as soon as an overall (allowing for the non-homogeneous mechanical properties of the polysilicon film) tensile strength of polysilicon is attained. Afterwards, instead of instantaneously annihilate the local load-carrying capacity (procedure that would lead to possible numerical instabilities and to a pathological mesh-dependence of the results), a description of the progressive formation of the traction-free crack is obtained through a smooth decay of the virgin, pre-cracking mechanical properties.

## 4. Brittle Cracking Model

Silicon fails under tensile loading by (micro-)cracking, see e.g., [28,29]. It is generally assumed that a material is brittle when the length of the PZ, where dissipative phenomena at the sub-micron length-scale eventually lead to the formation of a traction-free crack, is small (negligible, in principle) if compared to the structural size. According to Irwin’s model [30], in silicon this PZ length amounts to about 20 nm [21], which is one order of magnitude smaller than the characteristic grain size of the polysilicon adopted in MEMS manufacturing, and two orders of magnitude smaller than the size of structural features of the movable parts of MEMS themselves. Therefore, the assumption of brittle response looks appropriate to capture the load-bearing capacity of the sensor as a whole; nonlinearities linked to distributed micro-cracking processes need instead to be accounted for at the micro-scale.

As said, constitutive modelling of polysilicon is obtained with a smeared-crack approach, already implemented in the commercial Abaqus finite element code (Simulia) [31]. According to the Rankine strength criterion [32], PZs can nucleate as soon as the maximum principal stress locally attains the tensile strength of silicon; hence, crack is assumed to be always nucleated in mode I [33]. Once a PZ is nucleated, the local fields are decomposed into elastic and inelastic (cracking) contributions; upon continuous loading, it is expected that the PZ opens and the elastic straining in the surrounding bulk accordingly decreases. To capture the strength reduction following the inception of micro-cracking (*i.e.*, the softening response), the resistance offered by the PZ is modelled as a cohesive-like, decreasing function of the displacement jump across the PZ itself.

Since the PZ and the traction-free crack are not explicitly allowed for by the smeared-crack approach, a cracking strain term (instead of the displacement jump) is handled in the region where the stress field already attained the inception threshold. This technique is known to lead to mesh-dependent results [32]; the drawback is here solved by linking the displacement jump to the cracking strain through a material-dependent characteristic length (called localization limiter, see e.g., [34]). This provision allows the fracture energy, *i.e.*, the energy to be dissipated in order to form a traction-free crack, to be an objective, mesh-independent parameter in the simulations.

Furthermore, since the above approach can lead to locking (*i.e.*, excessively stiff results) under some loading conditions, the shear elastic moduli are assumed to be a decreasing function of the local displacement jump, till annihilation once a traction-free crack is formed.

Even if the aforementioned provisions are adopted to get objective outcomes, it may happen that results are still affected by the space discretization; this occurs if the characteristic size of the elements is not small enough to accurately resolve the stress and strain fields inside the PZ. In Section 5 below we therefore investigate this numerical issue, in order to ensure attainment of objective failure indicators.

## 5. Two-Scale Analysis of a Uni-Axial Accelerometer Subjected to Drops

In this Section we investigate the effect of drops on the device depicted in Figure 7, and we assess the capabilities of the offered two-scale numerical approach to reliably simulate the relevant failure mechanisms. The geometry of the whole package was already described in details in [22]. In our investigation we consider all the materials constituting the package (support plate, die-cap and ASIC, mould) to behave elastically, and to be perfectly joined together. The only foreseen failure mechanism is therefore linked to the cracking of the movable parts of the MEMS sensor.

Concerning the overall (meso-scale) elastic properties of the polysilicon film, allowing for its columnar grain morphology [35] a transversally isotropic symmetry is assumed, with the axis of transverse isotropy aligned with the normal to the substrate. In the reference frame depicted in Figure 7(b), the elastic moduli read: *E_x_* = *E_y_* = 150 GPa, *E_z_* = 130 GPa, ν*_xy_* = 0.2, ν*_xz_* = ν*_yz_* = 0.28, *G_xz_* = *G*_yz_ = 80 GPa [18,36]. To model crack nucleation and evolution, the overall strength and toughness of polysilicon are deterministically assumed equal to τ_M_ = 3.8 GPa and ϕ = 7 J/m^2^, respectively [14,19,21]. Because of the weak anisotropy of the elastic, strength and toughness properties of single-crystal silicon (see, e.g., [36–39]), meso-scale analyses accounting for the aforementioned homogenized, in-plane isotropic mechanical properties of polysilicon are expected to provide accurate outcomes.

Figures in Section 2 showed that polysilicon outer surfaces are not flat, because of the microstructure. In the analyses to follow we have instead assumed the surfaces of the whole sensor to be perfectly flat, thereby disregarding the actual GB geometry; this simplifying assumption is not affecting the meso-scale results, since the homogenized mechanical properties introduced above account for the overall effects of the granular microstructure.

We assume that the packaged sensor strikes the ground (*i.e.*, the target surface) after a free fall from a height of 2.5 m. In [20,21] we showed that, independently of the falling orientation, this drop height almost surely (within a stochastic framework) leads to sensor failure.

The aim of the remainder of this Section is three-fold. First, we show that meso-scale outcomes are objective, *i.e.*, that through mesh refinement we converge toward space discretization-independent failure descriptions. Second, we validate the proposed two-scale approach against full three-scale simulations (which allow for polysilicon morphology features), showing that both approaches foresee the same failure mechanisms. Third, we investigate the links between drop orientation, failure mechanism and time to failure.

### 5.1. Effects of Space Discretization

**Figure 8.**
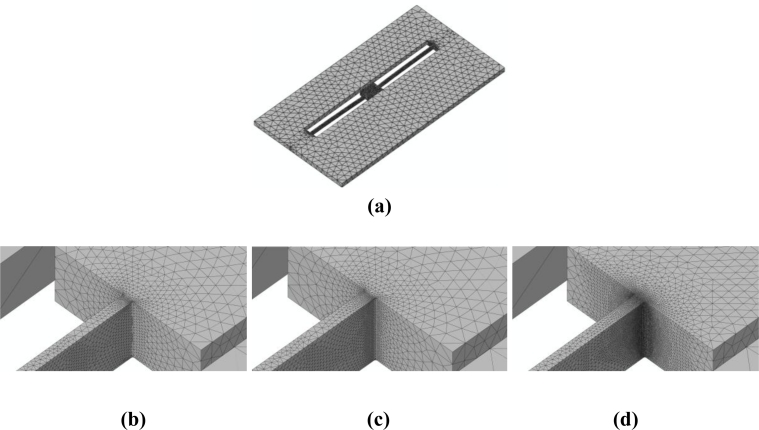
Adopted space discretizations: **(a)** overview of whole discretization; closed-up views of the meshes featuring **(b)** *d_e_* = 200 nm, **(c)** *d_e_* = 150 nm, and **(d)** *d_e_* = 100 nm at the anchor-suspension spring connection.

To assess the effects of mesh refinement on the predicted failure mechanism, we handle three values of the characteristic element size *d_e_* in the failing region, *i.e.*, at the connection between the MEMS anchor and suspension springs: *d_e_* = 200 nm [Figure 8(b)], *d_e_* = 150 nm [Figure 8(c)], and *d_e_* = 100 nm [Figure 8(d)]. As depicted in Figure 8, the space discretizations are purposely refined much only within the regions expected to fail, according to previous investigations [18–22]; out of these regions, the MEMS is instead coarsely meshed to reduce the computational burden. The aforementioned values of *d_e_* are all much higher than that necessary to achieve accuracy in the description of crack propagation at the micro-scale, which amounts to *d_e_* = 10 nm [21].

To investigate mesh-dependence, we consider two drop orientations, here termed bottom and top ones; device surfaces striking the target in the two falling cases are shown in Figure 7(a). The former orientation is characterized by the package striking the flat target with its own bottom surface, whereas the latter orientation is characterized by the package falling up-side down and striking the target with its top surface. In both cases, plane waves start propagating inside the package after the impact; these waves are thereafter partially reflected and dispersed by inner surfaces bonding different materials, and eventually impinge upon the sensor anchor. The resulting loadings on the MEMS turn out to be greatly different in the two cases, because of the different elastic properties of the package material striking the target, and because of the different paths followed by the stress waves from the contact point to the MEMS anchor, see [22]. A further role to define failure is played by the different gap between the seismic plate and the surfaces of die and cap, which respectively supports and insulates the sensor from the outer environment. Since the gap between plate and die is less than the gap between plate and cap, a scattered shock-carrying capacity of the sensor was reported in [22].

**Figure 9.**
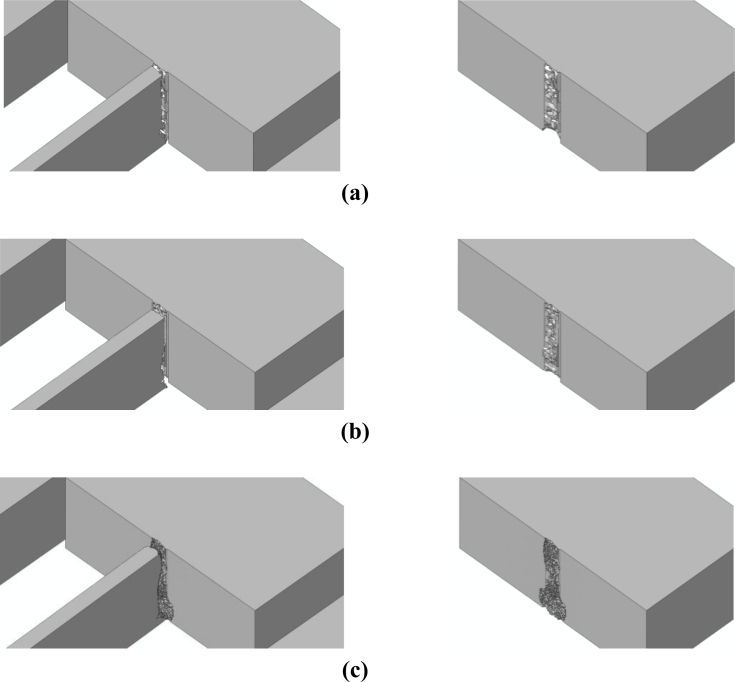
Bottom drop; forecasted crack pattern: **(a)** *d_e_* = 200 nm; **(b)** *d_e_* = 150 nm; **(c)** *d_e_* = 100 nm.

**Figure 10.**
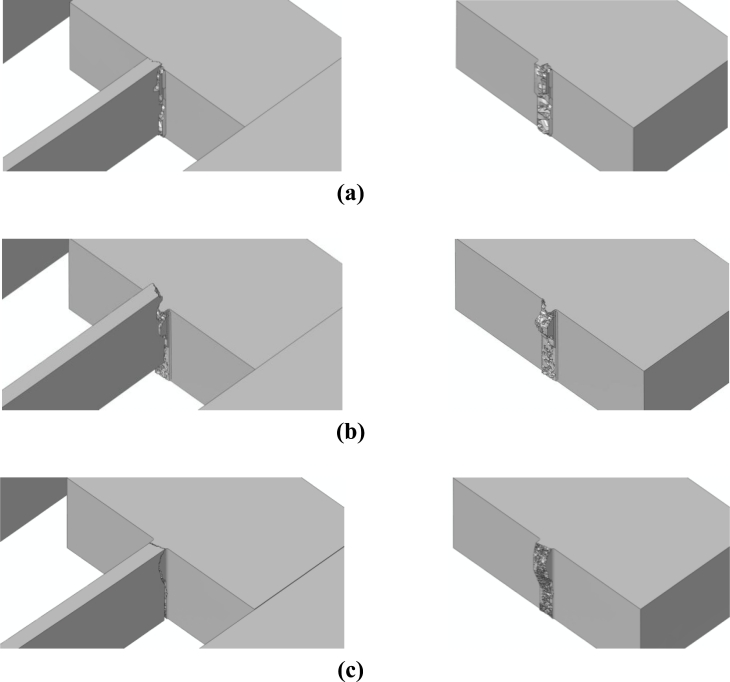
Top drop; forecasted crack pattern: **(a)** *d_e_* = 200 nm; **(b)** *d_e_* = 150 nm; **(c)** *d_e_* = 100 nm.

The effects of *d_e_* on the forecasted drop-induced failure mechanisms are shown in Figures 9 and 10 in the case of bottom and top drops, respectively. These figures depict the whole sensor when crack percolation occurs (*i.e.*, at complete failure of a cross-section of the suspension springs); to get insights into the geometry of the failure loci, the right columns of the figures show only the MEMS anchor, and hence one side of the resulting part-through crack.

**Figure 11.**
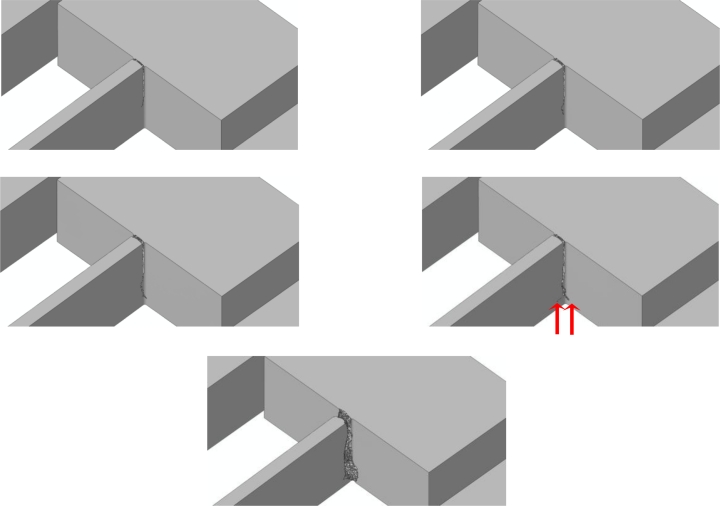
Bottom drop, *d_e_* = 100 nm: forecasted crack evolution.

**Figure 12.**
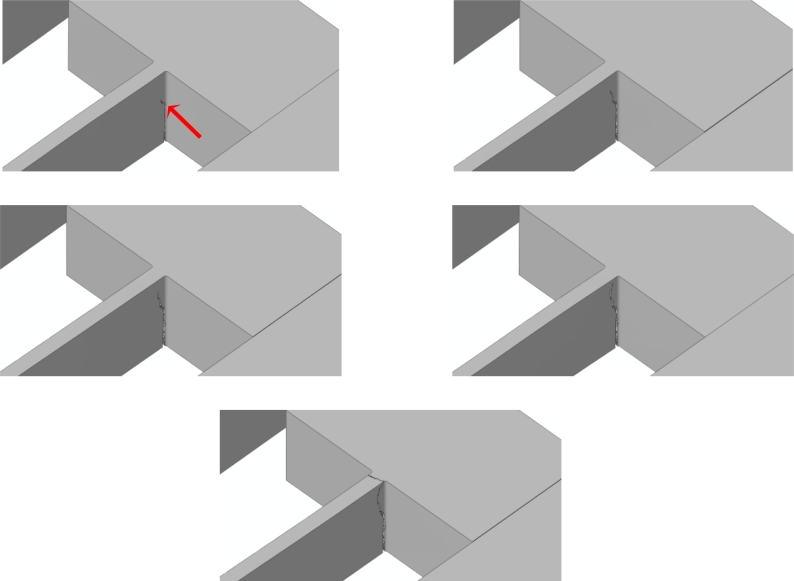
Top drop, *d_e_* = 100 nm: forecasted crack evolution.

Because of the re-entrant corners and the spring vibrations induced by the impact, it happens that crack starts propagating very close to the spring-anchor connection, at the top (bottom) free surface in case of a bottom (top) drop. This is clearly depicted in Figures 11 and 12, which gather the two evolving crack patterns, as obtained with *d_e_* = 100 nm. The spring dynamics also affects the crack evolution, leading to branching in the bottom drop case (marked with the two arrows in Figure 11) and to kinking in the top drop case (marked with the arrow in Figure 12).

All these outcomes testify that the location of crack initiation does not depend on the adopted mesh, being determined by the loading conditions only. On the other hand, mesh refinement allows one to obtain a better description of the crack path, till percolation: crack faces appear smoother and smoother by decreasing *d_e_*. As far as the characteristic times of failure are concerned, crack inception respectively occurs 1.16 μs and 1.77 μs after the impact in the bottom and top drops. The whole crack event due to the bottom drop, up to percolation, takes place in 0.13–0.14 μs, independently of the mesh. Crack percolation due to the top drop instead occurs within 0.05 μs, 0.11 μs and 0.13 μs in the simulations featuring *d_e_* = 200 nm, *d_e_* = 150 nm and *d_e_* = 100 nm; hence, time to failure tends to converge toward an (almost) element size-independent value upon mesh refinement.

A validation of the results presented in [22] is eventually obtained: independently of the drop configuration, failure occurs in the region already located by linear elastic analyses (without any provision to model crack inception and growth). The resulting failure mechanisms well agree with the available experimental outcomes depicted in Figure 2: failure is correctly located close to the connection between slender and massive parts (see Section 2), basically due to the stress amplification caused by the re-entrant corners. It needs to be remarked that numerical and experimental results do not perfectly match, since the failure mechanism depicted in Figure 2 was obtained after a free (not guided) fall of a device similar to that here analyzed, without a control of the drop orientation.

### 5.2. Validation through Comparison with a Three-Scale Monte-Carlo Approach

**Figure 13.**
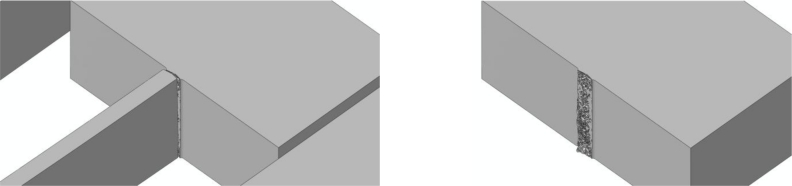
Failure mechanism induced by the bending-dominated loading (side-A drop).

**Figure 14.**
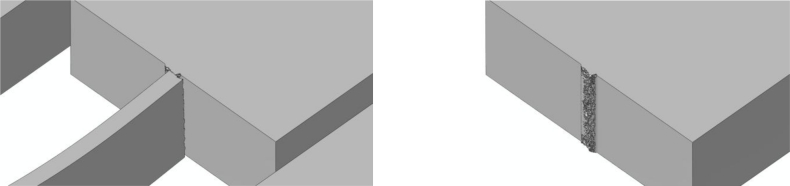
Failure mechanism induced by the tension-dominated loading (side-B drop).

**Figure 15.**
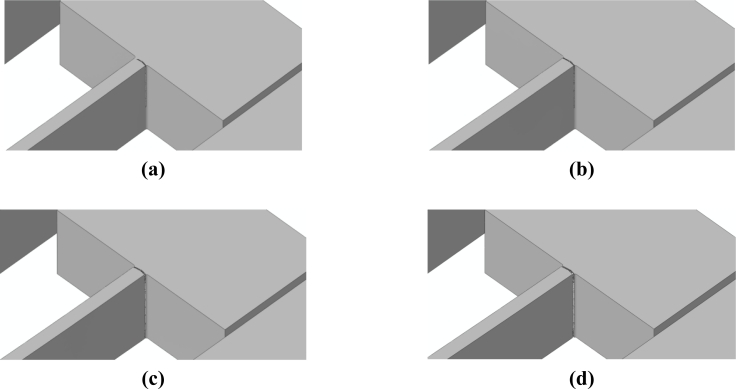
Side-A drop, forecasted crack patterns at time **(a)** *t* = 1.18 μs, **(b)** *t* = 1.19 μs, **(c)** *t* = 1.20 μs, and **(d)** *t* = 1.25 μs.

**Figure 16.**
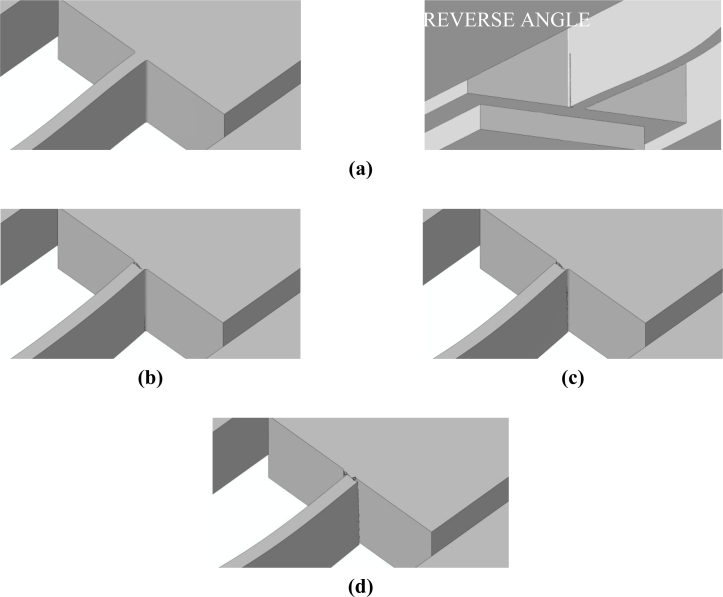
Side-B drop, forecasted crack patterns at time **(a)** *t* = 0.77 μs, **(b)** *t* = 0.78 μs, **(c)** *t* = 0.79 μs, and **(d)** *t* = 0.81 μs.

Having assessed the objectivity of the proposed two-scale approach, we now investigate two alternative drop configurations that do not induce a torsional deformation in the suspension springs, see [21]. These two drops are characterized by the package striking the target with side A or B, see Figure 7(a): because of inertial effects, the former drop induces a bending-dominated state of stress in the springs, whereas the latter drop induces a tension-dominated stress state in the failing spring [21]. The forecasted crack paths induced by the bending- and tension-dominated loadings are respectively shown in Figures 13 and 14. Figures 15 and 16 instead show the evolution of the failure mechanisms in the two cases.

These drop configurations were recently investigated in [21]; the effect of the polysilicon morphology on failure was assessed there through a three-scale approach, wherein the outcomes at the micro-scale were obtained by stochastically handling the crack patterns furnished by Monte-Carlo simulations. Since the polysilicon morphology cannot be known deterministically at this length-scale, in micro-scale simulations we assumed the GB geometry (explicitly modelled) and the orientation of the axes of elastic symmetry of each silicon grain to be random fields, allowed to change in each analysis.

Crack inception is predicted by the two- and three-scale approaches to occur at (almost) the same drop case-dependent time instant. In [21], crack percolation at the spring-anchor connection was reported to occur in about 0.013 μs, both under tension- and bending-dominated loadings. The present two-scale analyses (see Figures 15 and 16) predict instead time intervals of about 0.04 μs and 0.07 μs to obtain a part-through crack; the cracks propagate almost instantaneously from side to side, along the suspension spring top or bottom surfaces [see Figures 15(a) and 16(a)], but they require the aforementioned time intervals to grow along the 15 μm-thick polysilicon film.

**Figure 17.**
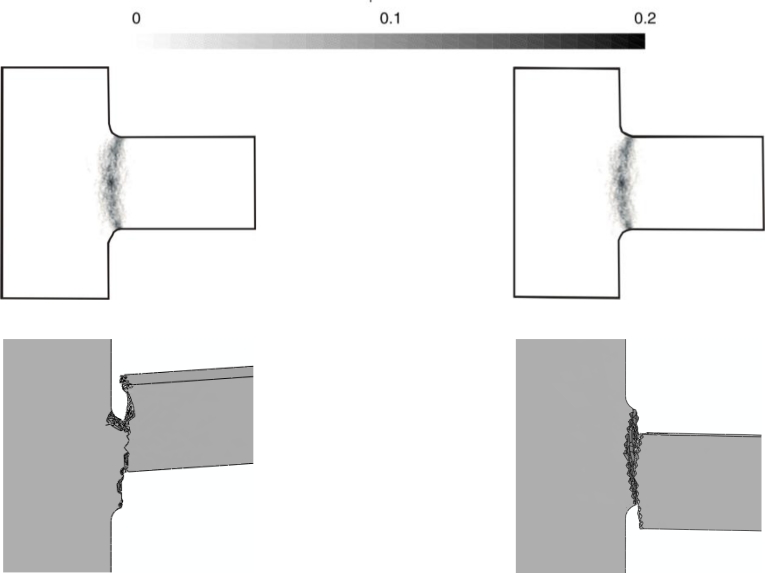
Comparison between (top row) three-scale and (bottom row) two-scale forecasts of the crack patterns at failure, in the case of (left column) tension-dominated loading, and (right column) bending-dominated loading.

A comparison of the failure mechanisms predicted by the present two-scale approach and by the three-scale approach of [21] is illustrated in Figure 17, for the two drop configurations. The probabilistic failure maps of the Monte-Carlo simulations (top row of Figure 17) have this meaning: a value equal to 0 means that, independently of the polysilicon microstructure, a crack would never pass through that point; on the contrary, a value equal to 1 means that the crack is surely nucleated at that point after the drop. It results that both approaches predict crack patterns well confined around the spring cross-section connected to the anchor, as also reported experimentally (see Figure 2).

## 6. Concluding Remarks

In this paper, we have presented a two-scale approach to model drop-induced failures of polysilicon MEMS sensors. To attain accuracy in the description of the failure mechanism at affordable computational costs, it has been shown that a multi-scale approach appears necessary to properly account for the several length-scales involved in failure process, ranging from millimetres (at the package level) down to nanometres (at the polysilicon film level).

In former papers we already proposed a similar two-scale framework, but we did not address the whole tracking of the failure mechanism. We also developed a three-scale approach, wherein uncertainties at the polysilicon level linked to its polycrystalline features were handled through a Monte Carlo methodology; this time-consuming approach has been used here as a benchmark to assess the accuracy of the offered two-scale simulations.

Results have shown that the proposed scheme provides objective forecasts of the failure mechanism, granted that the space discretization is fine enough in the failing region(s). The required characteristic element size has been shown to amount to *d_e_* = 100 – 150 nm, whereas it amounts to *d_e_* = 5 – 10 nm to attain accuracy in the micro-scale simulations used for benchmark purposes. Since in the present two-scale analyses only the overall mechanical properties of polysilicon need to be accurately provided (while in the three-scale analyses the local polysilicon properties have to be simulated within a Monte Carlo procedure), a huge saving of computational costs is obtained. On the other hand, discrepancies in the forecasted failure mechanisms look negligible.

The present two-scale framework therefore appears attractive from an industrial perspective, owing to the good performance and accuracy attained without a deep knowledge of the actual polysilicon microstructure in the failing region.
